# Effects of dietary fat subtypes on glucose homeostasis during pregnancy in rats

**DOI:** 10.1186/s12986-016-0117-7

**Published:** 2016-08-24

**Authors:** Len H. Storlien, Yan Y. Lam, Ben J. Wu, Linda C. Tapsell, Arthur B. Jenkins

**Affiliations:** 1 Boden Institute of Obesity, Nutrition, Exercise & Eating Disorders and Charles Perkins Centre, University of Sydney, Sydney, NSW 2006 Australia; 2 Metabolic Research Centre, University of Wollongong, Wollongong, NSW 2522 Australia; 3 School of Medicine, Smart Foods Centre, Illawarra Health and Medical Research Institute, University of Wollongong, Wollongong, NSW 2522 Australia

**Keywords:** Pregnancy, Dietary fat, Insulin sensitivity, Hyperinsulinemia

## Abstract

**Background:**

Dietary n-3 and n-6 polyunsaturated fatty acids (PUFAs) have an impact on insulin secretion and sensitivity but whether and how these may be related to maternal glucose homeostasis during pregnancy is unclear.

**Methods:**

Female Wistar rats (240–250 g) were assigned to laboratory CHOW or high fat diets rich in either n-6 (safflower oil; n-6 group) or n-6 + n-3 (safflower oil + fish oil; n-3 group) PUFAs. After 10 days half of the animals in each diet group were inseminated and confirmed pregnant. An overnight fasted intravenous glucose tolerance test (500 mg glucose/kg body weight) was performed on chronically cannulated non-pregnant and 20-day pregnant rats. Indices of insulin secretion (β) and insulin sensitivity (S) were calculated from the plasma glucose and insulin responses. The fatty acid composition of phospholipids was determined in samples of liver and two skeletal muscles (soleus and red quadriceps).

**Results:**

Pregnancy in the CHOW group significantly increased β (*P* < 0.001) and decreased S (*P* < 0.01). In contrast, both n-6 and n-3 diets abolished both the pregnancy-induced decrease in S and pregnancy-induced increase in β with the n-3 diet having a more potent effect on both S and β. S was positively correlated with the sum of n-3 fatty acids, with docosahexaenoic acid (22:6 n-3) the major contributor, in liver (*r* = 0.485; *P* < 0.001), red quadriceps (*r* = 0.421; *P* = 0.004) and soleus (*r* = 0.476; *P* < 0.001). In contrast S was inversely related to arachidonic acid (20:4n-6) levels in liver and red quadriceps across all groups and these relationships were particularly powerful in pregnancy (liver: *r* = -0.785; red quadriceps: *r* = -0.754, both *P* < 0.0001).

**Conclusions:**

The results demonstrate potent effects of dietary fat amount and profile on glucoregulation during pregnancy and emphasize the importance of the balance between dietary n-3 and n-6 PUFAs.

**Electronic supplementary material:**

The online version of this article (doi:10.1186/s12986-016-0117-7) contains supplementary material, which is available to authorized users.

## Background

Gestational diabetes mellitus (GDM) can be defined as carbohydrate intolerance of varying severity with onset or first recognition during pregnancy [1]. In addition to increasing the risk of complications during pregnancy and at delivery [2], women with a history of GDM are more likely to develop type 2 diabetes in the future and their offspring are also at a greater risk of developing childhood obesity and other adverse metabolic outcomes [3]. Precisely how pregnancy leads to GDM is still not entirely clear. Normal pregnancy is characterized by a progressive increase in insulin resistance, associated with β-cell hyperplasia and a large increase in insulin secretion, that leads to a transient state of glucose intolerance [1]. Certain metabolic and hormonal stresses during pregnancy, however, appear to create an extreme manifestation of metabolic alternations that results in GDM [4].

Dietary fat consumption, specifically the balance of fatty acid subtypes, are likely to play a major role in optimal maternal and fetal health as there is an increasing delivery of long-chain polyunsaturated fatty acids (PUFAs) ramping up later in pregnancy to supply, *inter alia*, docosahexanoic acid (22:6n-3; DHA) critical for brain and nervous system development [5, 6]. These PUFAs must come from the diet of the mother and their amount and the n-3/n-6 balance may well be one of the “stressors” that drives the pathogenesis of GDM [7, 8]. There is already a good deal of evidence from non-pregnant humans and experimental animal models showing that dietary fatty acid composition strongly influences both insulin secretion and sensitivity [9–11]. While the majority of the literature also support increased saturated fat intake as an independent risk factor for GDM or glucose intolerance during pregnancy [12–14], the role of long-chain PUFAs in the context of glucose homeostasis of pregnant women is less clear [13, 15]. Reliable consumption data on PUFA subtypes (n-3 vs n-6) are scarce and the materno-fetal transfer of n-3 PUFAs, especially DHA for fetal development of the central nervous system, further complicates the relationship between dietary intake, bioavailability and effects on maternal glucoregulation.

We therefore aimed to investigate the effects of varying dietary total fat, and n-3 and n-6 PUFA intake on intravenous glucose tolerance in pregnant rats using derived measures of insulin secretion and systemic insulin action, with a specific focus on fatty acid composition in insulin-sensitive tissues a potential key variable underlying the relationship between dietary PUFA intake and maternal glucose homeostasis.

## Methods

### Animals

Female Wistar rats (12 weeks old at the beginning of the study) were obtained from the Australian Animal Resources Centre (Perth, Australia). All animals were housed individually in the animal facility of the University of Wollongong and in a temperature-controlled environment (22 °C) with a 12-h light/dark cycle. Following 1 week of acclimatization, animals were randomized into 3 diet groups (see below) with 2 subgroups per diet, pregnant and non-pregnant. After receiving their corresponding diets for 10 d, animals in the pregnant group were individually placed with a single sexually mature male rat for a period of 2–3 d, during which they were checked at 12-h intervals for evidence of insemination (vaginal plug). The day on which a vaginal plug was found was designated as day 1 of pregnancy. This procedure resulted in 100 % successful insemination. After insemination, females returned to their individual cages and their dietary treatment continued. Cannulation was performed at day 15 of pregnancy for pregnant rats and age-matched virgin rats as control. Rats were anaesthetized with an intramuscular injection of 30 mg/kg body weight Rompun (xylazine) and 50 mg/kg body weight Ketamine. Cannulas were inserted in the right and left jugular veins to infuse glucose and withdraw blood during the intravenous glucose tolerance test [16].

### Diets

The 3 experimental diets were: a standard low fat/high carbohydrate laboratory chow (5 % energy from fat from a combination of lard, safflower and canola oils; Y.S. Feeds, Young, Australia; CHOW), a low carbohydrate/high n-6 PUFAs diet (59 % energy from fat, of which 100 % was safflower oil; n-6 group) and a low carbohydrate/high n-3 PUFAs diet (59 % energy from fat, of which 30 % was fish oil (Max EPA, Scherer, Melbourne, Australia) and 70 % was safflower oil; n-3 group). The high fat diets were prepared in-house [17], made freshly every 3–4 d and stored at 4 °C until use. Each animal was given 310 kJ/day which was approximately their *ad libitum* intake of laboratory chow at study entry to avoid major changes in caloric intake and obesity. Spillage, if any, was collected and additional diet of equal weight was added to the next day’s intake. Overall, all rats were observed to eat all the food provided over the course of the experiment. Animals were fed on their respective diets for a total of 5 weeks.

### Intravenous glucose tolerance test (IVGTT)

At the end of 5 d post-cannulation (day 20 of pregnancy for pregnant rats), rats were fasted overnight and allowed at least 30 min acclimatization in testing cages prior to the test. Immediately after taking a baseline blood sample (400 μl), rats received an intravenous bolus of 1 ml/kg body weight glucose (50 % w/v) over a 10-s interval. Blood samples (200 μl) were taken at 2, 5, 10, 15 and 30 min and centrifuged at 12,000 × g for 1 min. Plasma were stored at -20 °C for not more than one month until assayed for glucose and insulin concentrations. The remaining blood cells from the baseline and 10-min samples were pooled and re-suspended in 9 g/L saline, 1 × 10^5^ U/L heparin to a total volume of ~ 600 μl. Re-suspended blood cells were infused into the animal between the 15- and 30-min blood draws to maintain body fluids and hematocrit levels. At the completion of the IVGTT, rats were killed by an intravenous overdose of Nembutal (120 mg/kg body weight). Samples of skeletal muscle and liver were snap frozen and stored at -80 °C for subsequent analysis.

### Biochemical analyses

Plasma glucose and insulin were measured in duplicates using commercially available assay kits from Roche (Mannheim, Germany) and EMD Millipore (St Charles, MO) respectively. The extraction and derivatization of the fatty acids in muscle and liver phospholipids have been described elsewhere [18, 19]. Briefly, tissue samples were homogenized in 2:1 (v/v) chloroform: methanol and total lipid extracts were isolated. Phospholipids and triglycerides were then separated by solid phase extraction on Sep-pak silica cartridges (Waters, Milford, MA). The phospholipid fraction was transmethylated with 140 g/L boron trifluoride (Sigma-Aldrich, St Louis, MO) at 85 °C for 1 h. Methyl esters were extracted into hexane. Following the removal of polar contaminants, the methyl fatty acids were separated, identified and quantitated by gas chromatography. The content of individual fatty acids in the phospholipids was expressed as a percentage of the total fatty acids. Liver triglycerides were analyzed as above except the dried triglycerides were re-dissolved in 200 μl of 95 % (v/v) ethanol. The amount of triglycerides was determined using an enzymatic colorimetric test kit with a Cobas Mira® biochemistry analyzer (Roche Diagnostics, Sydney, Australia).

### Insulin secretion and insulin sensitivity indices

The submaximal sensitivity of insulin secretion to glucose (β) was calculated from the areas under the curves (AUCs) of the plasma glucose and insulin responses to the IVGTT as InsulinAUC_0-30_/GlucoseAUC_0-30_ [20]. An index of insulin sensitivity (S) was obtained under the assumption that the integrated responses (AUC) to an IVGTT can be treated as a quasi-steady state and can therefore be explained by a simple homeostatic model assessment-like model [21]. Under the conditions tested (fasting, glucose clamp, IVGTT) any two of glycemia, the glucose-sensitivity of insulin secretion, and insulin sensitivity can be used to calculate the third using the following equation:

$$ G=\frac{R_{in}}{\beta \kern0.5em \cdotp \kern0.5em S} $$

where *G* = glucose concentration, *R*_*in*_ = the rate of entry of glucose into the system, β = the glucose-sensitivity of insulin secretion, and *S* = the insulin sensitivity of glucose disposal.

The index S was derived from this relationship as 1/(β* GlucoseAUC_0-30_) which, substituting for β becomes 1/InsulinAUC_0-30_. In this study *R*_*in*_ is composed of the known amount of exogenous glucose delivered intravenously plus an unknown amount of residual hepatic glucose output. As *R*_*in*_ was ignored in the calculation of S assuming that the rate of entry was constant across animals, the index will contain any effects of treatments on endogenous glucose output during IVGTT as well as effects on S. It will also contain any effects of treatment on non-insulin sensitive glucose disposal, which is omitted from the model. The increased glucose uptake by the fetal-placental unit will, to the extent that it is insulin-insensitive, lead to overestimation of S during pregnancy. Using the glucose turnover estimates obtained in pregnant rats by Nolan and Proietto [22], and assuming that all of the additional glucose uptake in pregnancy is insulin-insensitive, we calculate that S will be overestimated in pregnancy by 20 %. It should be noted that glucose uptake by the fetal-placental unit might be affected by litter size and total fetal weight. However we did not note any effect of dietary composition or fat content on litter size. This appears to be consistent with the bulk of the relevant literature [23, 24]. This degree of overestimation due to any fetal drain does not affect any conclusions drawn from the analyses of S. The index β contains any effects of treatments on insulin clearance as well as effects on insulin secretion.

### Statistical analyses

All summary data are presented as mean ± SEM. Effects of pregnancy and diet were analyzed using two-way ANOVA including an interaction term (Pregnancy*Diet) with Bonferroni post hoc tests to compare group differences. Relationships between continuous variables were analyzed using Pearson correlation. All analyses were performed using JMP software (version 4.0.1, SAS, Cary, NC) and GraphPad Prism Program (version 6.04, GraphPad Software, San Diego, CA). Significance was accepted at *P* value < 0.05.

## Results

### Body weight

All groups were matched for body weight at entry to the study (Table 1). After 5 weeks of diet treatments, there was the expected increase in body weight associated with pregnancy across all diet groups (*P* < 0.001). Diet also had an independent effect on body weight (*P* < 0.001) with animals in the n-6 group significantly heavier (*P* < 0.05) than those in the CHOW group and no difference between the n-3 and n-6 groups (Table 1).

**Table 1:** Body weight and biochemical analyses (*N* = 7–8 per group)

	CHOW		N-3		N-6		*P*-value		
	Non-pregnant	Pregnant	Non-pregnant	Pregnant	Non-pregnant	Pregnant	Pregnancy	Diet	Interaction
Before dietary interventions									
Weight [g]	241 ± 3	242 ± 2	245 ± 3	242 ± 1	246 ± 3	246 ± 4	NS	NS	NS
After dietary interventions									
Weight [g]	265 ± 3	303 ± 5	284 ± 2	319 ± 4	287 ± 3	319 ± 2	<0.001	< 0.001	NS
Fasting glucose [mmol/L]	5.78 ± 0.25	4.41 ± 0.12	6.54 ± 0.52	3.91 ± 0.33	6.83 ± 0.53	6.01 ± 0.34	< 0.001	< 0.01	NS
Fasting insulin [pmol/L]	161 ± 26	280 ± 52	189 ± 36	116 ± 19	162 ± 28	305 ± 45	< 0.05	NS	< 0.01
IVGTT glucose AUC[min*mmol/L]^a^	270 ± 20	183 ± 6	300 ± 23	219 ± 12	325 ± 18	260 ± 12	< 0.001	< 0.01	NS
IVGTT insulin AUC[min*nmol/L]^a^	11.9 ± 1.4	27.2 ± 3.9	10.3 ± 0.7	9.4 ± 1.1	17.0 ± 2.7	18.9 ± 2.9	< 0.01	< 0.001	< 0.01
Liver triglycerides [mmol/mg]	8.30 ± 0.69	7.79 ± 0.56	6.45 ± 0.89	6.13 ± 0.70	10.7 ± 0.89	8.31 ± 1.00	NS	< 0.001	NS

^a^ *IVGTT* intravenous glucose tolerance test, *AUC* area under the curve

### Glucose homeostasis

**Fig. 1:**
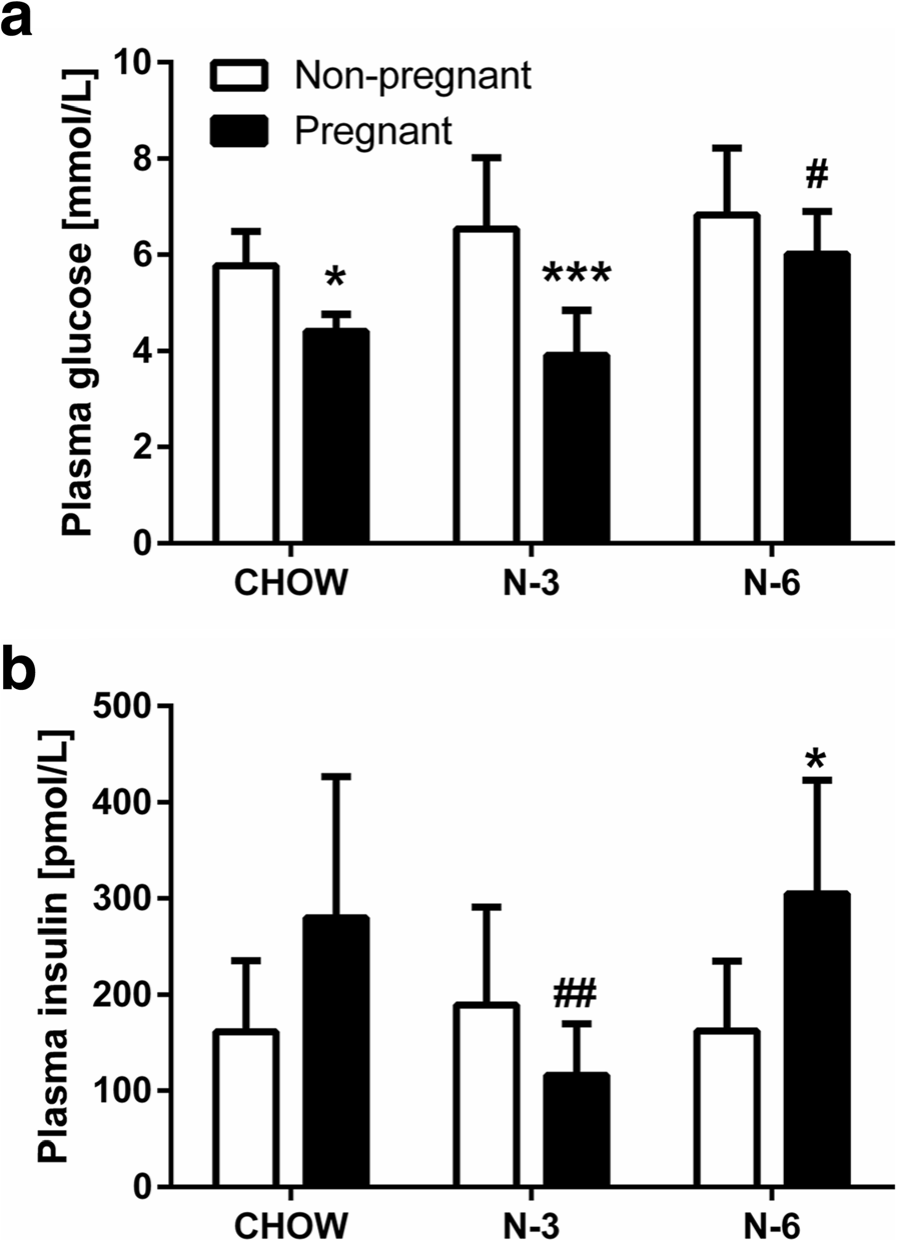
Effects of pregnancy and diet on fasting plasma glucose (**a**) and insulin (**b**) levels. *N* = 7–8 per group. *** *P* < 0.001 and * *P* < 0.05 compared to non-pregnant rats in the same diet group. ## *P* < 0.01 and # *P* < 0.05 compared to pregnant rats in the CHOW group. CHOW, standard laboratory chow diet; N-3, high n-3 long-chain polyunsaturated fatty acids diet; N-6, high n-6 long-chain polyunsaturated fatty acids diet

Fasting circulating levels of glucose and insulin did not differ between diet groups in non-pregnant rats (Fig. 1a and b). Consistent with the theory of a lower glucose “set point” during pregnancy [22], chow-fed pregnant rats had lower plasma glucose (4.14 ± 0.12 vs 5.78 ± 0.25 mmol/L for non-pregnant; *P* < 0.05) and higher plasma insulin (280 ± 52 vs 161 ± 26 pmol/L for non-pregnant; *P* = 0.07) concentrations. Pregnancy led to a similar reduction in fasting glucose in the n-3 group (*P* < 0.001; Fig. 1a) without altering the insulin level (Fig. 1b). In contrast, pregnancy was without effect on fasting glucose in the n-6 group despite a significant increase in insulin concentration (*P* < 0.05) comparable to that in pregnant CHOW rats (Fig. 1b).

**Fig. 2:**
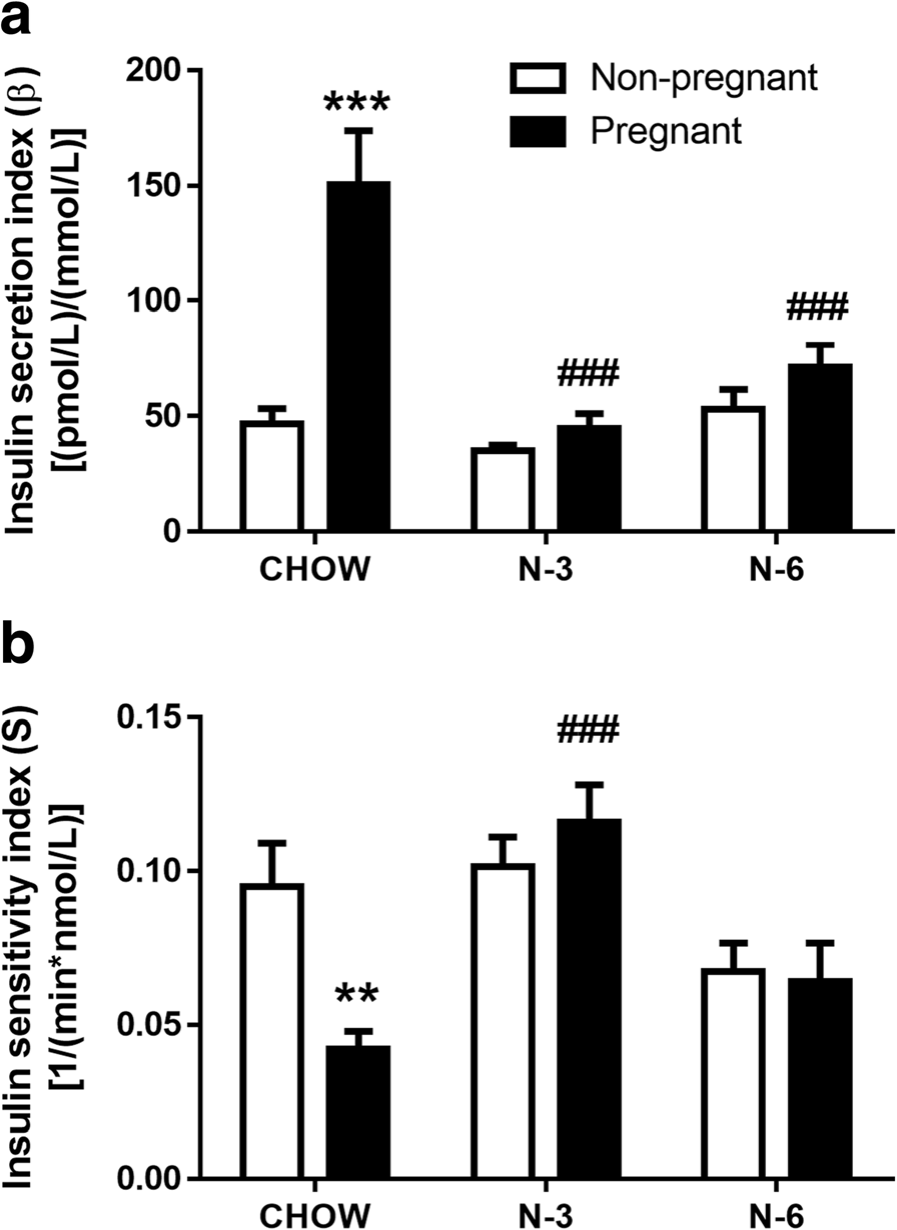
Effects of pregnancy and diet on glucose homeostasis in response to an acute glucose challenge. Insulin secretion (β; **a**) and insulin sensitivity (S; **b**) indices were calculated from the area under the curves of plasma glucose and insulin concentrations during the intravenous glucose tolerance test. *N* = 7–8 per group. *** *P* < 0.001 and ** *P* < 0.01 compared to non-pregnant rats in the same diet group. ### *P* < 0.001 compared to pregnant rats in the CHOW group. Diet abbreviations as per Fig. 1

In response to IVGTT, pregnancy lowered the circulating glucose across all diet groups (*P* < 0.05) but both the n-3 and n-6 diets negated the pregnancy-induced increase in plasma insulin levels (Table 1). We derived the insulin secretion (β) and insulin sensitivity (S) indices from the IVGTT responses to gain further insights into the dynamics of glucose homeostasis. Pregnancy induced a 223 % increase in β (*P* < 0.001) and a 56 % reduction in S (*P* < 0.01) in the CHOW group, effects that were completely abolished by either the n-3 or the n-6 diet (Fig. 2). Rats fed with a diet high in n-3 PUFAs also had the highest S among all pregnant groups (*P* < 0.01; Fig. 2b). Correction for the estimated bias in S during pregnancy reduced pregnant S by 20 % (see Methods) but had no effects on this analysis (data not shown), thus preserving the effect of both high fat diets on the response of S to pregnancy.

### Membrane lipid composition

**Fig. 3:**
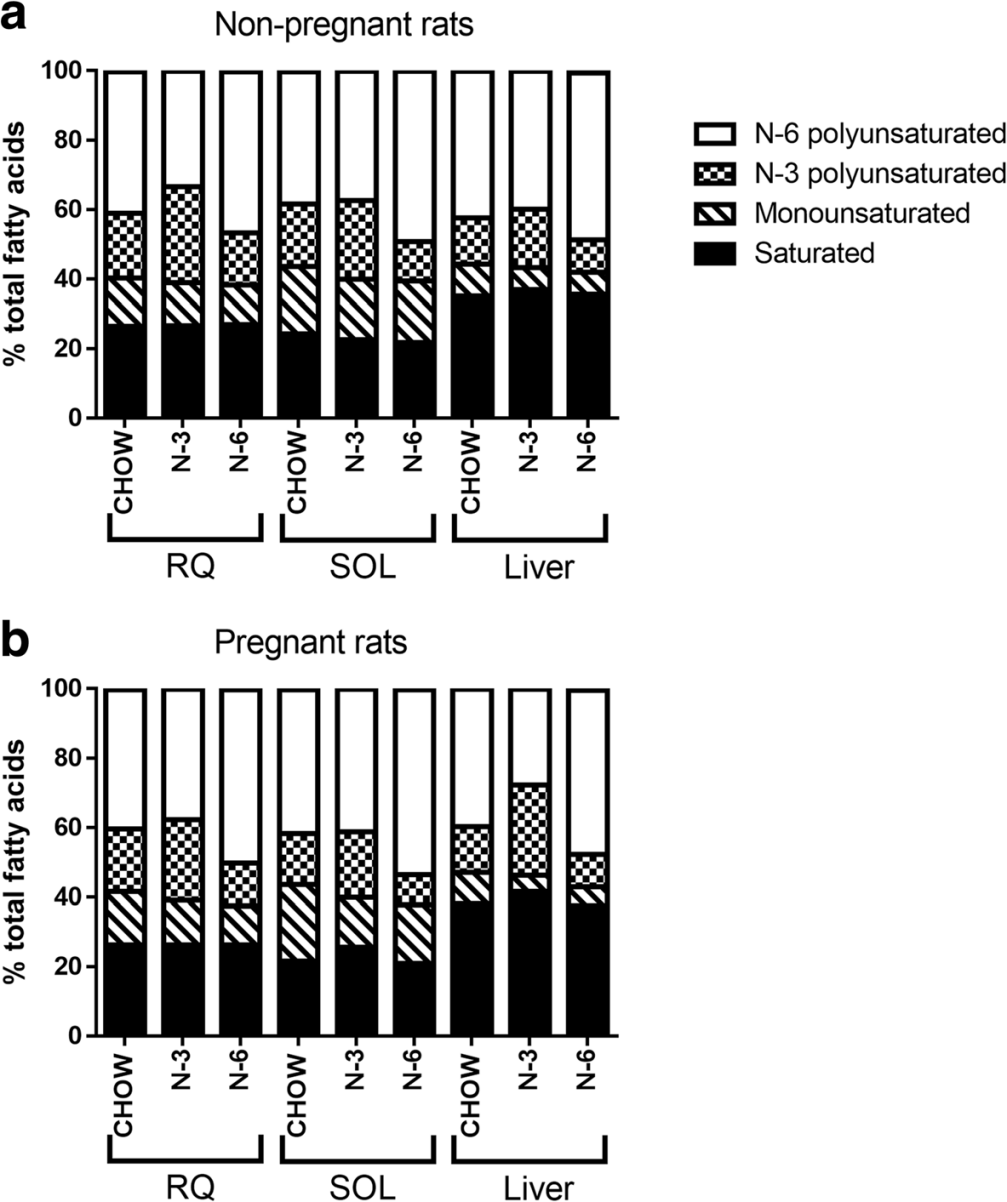
Effects of diet on membrane lipid composition in non-pregnant (**a**) and pregnant (**b**) rats. The abundance of fatty acid subtypes in tissue phospholipid was quantified using gas chromatography and expressed as percentage of total fatty acids. *N* = 7–8 per group. RQ, red quadriceps muscle; SOL, soleus muscle. Diet abbreviations as per Fig. 1

The fatty acid compositions of red quadriceps and soleus skeletal muscles and liver were profiled. In non-pregnant rats, the high fat diets had the expected effects on enriching the membrane lipids, with the proportion of the corresponding PUFAs increased at the expense of monounsaturated and/or other PUFAs whereas the proportion of saturated fatty acids remained unchanged (Fig. 3a). As a result, the n-3 group had a higher total n-3/n-6 ratio, and vice versa for the n-6 group (fatty acid profiles of muscle and liver in individual animal were included in Additional file 1: Table S1).

Pregnancy had no major effects on the PUFAs in muscle and liver in the CHOW group, except a significant reduction in n-3 PUFAs (14.6 ± 0.5 vs 17.9 ± 0.2 % for non-pregnant; *P* < 0.001) and an almost reciprocal increase in n-6 PUFAs (41.3 ± 0.6 vs 38.0 ± 0.8 % for non-pregnant; *P* < 0.001) in the soleus muscle. In rats fed with a high n-3 PUFAs diet, however, pregnancy induced tissue-specific changes in n-3 and n-6 PUFAs content. While there were more n-3 PUFAs (26.0 ± 0.7 vs 16.7 ± 0.9 % for non-pregnant; *P* < 0.001) and less n-6 PUFAs (27.6 ± 0.3 vs 39.7 ± 1.3 % for non-pregnant; *P* < 0.001) in the liver compared to their non-pregnant counterparts, the opposite trend (i.e., less n-3 PUFAs and more n-6 PUFAs) was observed in both the red quadriceps (*P* < 0.001) and soleus (*P* < 0.01; Fig. 3b). Overall, fatty acid unsaturation index (number of double bonds per fatty acid moiety) was significantly increased in red quadriceps and soleus muscles and trended to be elevated in the liver of pregnant rats fed with a high n-3 diet (*P* = 0.05). Despite a significant pregnancy-induced increase in the proportion of n-6 PUFAs in the red quadriceps (49.9 ± 0.6 vs 46.3 ± 2.1 % for non-pregnant; *P* < 0.05) and soleus (53.1 ± 0.6 vs 48.9 ± 1.6 % for non-pregnant; *P* < 0.01), membrane fatty acid profile of skeletal muscle and liver, as well as the fatty acid unsaturation index, in the n-6 group remained largely stable irrespective of pregnancy status.

### Relationships between membrane lipid composition and insulin sensitivity

**Fig. 4:**
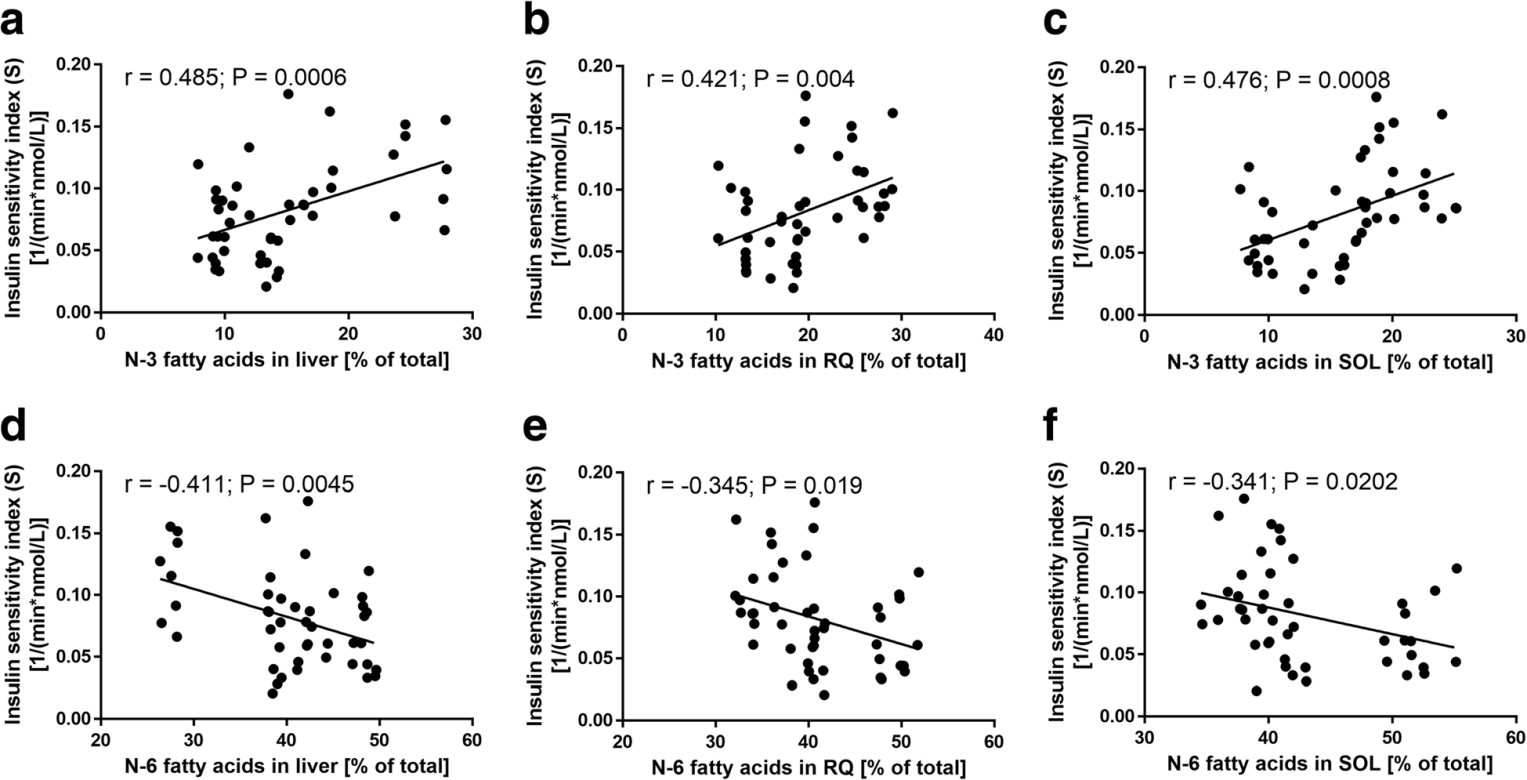
Correlations between tissue phospholipid composition and insulin sensitivity. **a**-**c**: correlations between the abundance of n-3 polyunsaturated fatty acids in liver (A), red quadriceps (**b**) and soleus (**c**) muscle with the insulin sensitivity index (S). **d**-**f**: correlations between the abundance of n-6 polyunsaturated fatty acids in liver (**d**), red quadriceps (**e**) and soleus (**f**) muscles with S. Rats from all diet and pregnancy groups were included in this analyses. *N* = 47. RQ, red quadriceps muscle; SOL, soleus muscle

Across all rats, irrespective of diet and pregnancy status, the proportion of n-3 PUFAs in the tissue phospholipid was positively correlated with S (*r* = 0.485 for liver, *P* < 0.001; *r* = 0.421 for red quadriceps, *P* = 0.004; *r* = 0.476 for soleus, *P* < 0.001; Fig. 4a–c). In contrast, there was an inverse relationship between the abundance of n-6 PUFAs in the membrane lipid with S (*r* = −0.411 for liver, *P* = 0.0045; *r* = −0.345 for red quadriceps, *P* = 0.019; *r* = −0.341 for soleus, *P* = 0.0202; Fig. 4d–f). Correlation statistics of individual treatment groups were summarized in Table 2.

**Table 2:** Correlations between tissue phospholipids and insulin sensitivity index

	All rats		Non-pregnant		Pregnant	
	r	*P*-value	r	*P*-value	r	*P*-value
Liver						
Sum of n-3 polyunsaturated fatty acids	0.485	0.0006	0.463	0.0259	0.625	0.0014
Sum of n-6 polyunsaturated fatty acids	−0.411	0.0045	−0.440	0.0357	−0.588	0.0032
Docosahexaenoic acid (22:6n-3)	0.454	0.0015	0.469	0.0241	0.628	0.0013
Arachidonic acid (20:4n-6)	−0.305	0.0394	−0.335	0.1180	−0.785	< 0.0001
Linoleic acid (18:2n-6)	0.283	0.0562	0.072	0.7434	0.454	0.0295
Red quadriceps						
Sum of n-3 polyunsaturated fatty acids	0.421	0.0036	0.357	0.0949	0.450	0.0314
Sum of n-6 polyunsaturated fatty acids	−0.345	0.0190	−0.360	0.0912	−0.291	0.1785
Docosahexaenoic acid (22:6n-3)	0.417	0.0039	0.344	0.1085	0.459	0.0274
Arachidonic acid (20:4n-6)	−0.532	0.0001	−0.284	0.1891	−0.754	< 0.0001
Linoleic acid (18:2n-6)	0.351	0.0168	−0.120	0.5851	0.635	0.0011
Soleus						
Sum of n-3 polyunsaturated fatty acids	0.476	0.0008	0.464	0.0256	0.451	0.0309
Sum of n-6 polyunsaturated fatty acids	−0.341	0.0202	−0.483	0.0195	−0.162	0.4611
Docosahexaenoic acid (22:6n-3)	0.468	0.0010	0.487	0.0183	0.410	0.0521
Arachidonic acid (20:4n-6)	0.476	0.0008	0.401	0.0640	0.451	0.0309
Linoleic acid (18:2n-6)	0.306	0.0386	−0.153	0.4850	0.662	0.0006

Next we examined the relationship between insulin sensitivity and individual fatty acids to gain functional insights. DHA made up to ~75–90 % of all n-3 PUFAs in tissues and, as expected, it was the major contributor to the overall n-3 PUFAs correlations (Table 2). With n-6 PUFAs we focused on arachidonic acid (20:4n-6) and its dietary precursor linoleic acid (18:2n-6), the two most predominant n-6 PUFAs in membrane phospholipid. The relationships between these two fatty acids and S were specific to tissue and pregnancy status. In the liver and red quadriceps of pregnant rats, arachidonic acid was negatively related to S (*r* = −0.785 for liver, *P* < 0.0001; *r* = −0.754 for red quadriceps, *P* < 0.0001), but the opposite was true for linoleic acid (*r* = 0.454 for liver, *P* = 0.0295; *r* = 0.635 for red quadriceps, *P* = 0.0011). Interestingly, none of these associations were observed in non-pregnant rats.

**Fig. 5:**
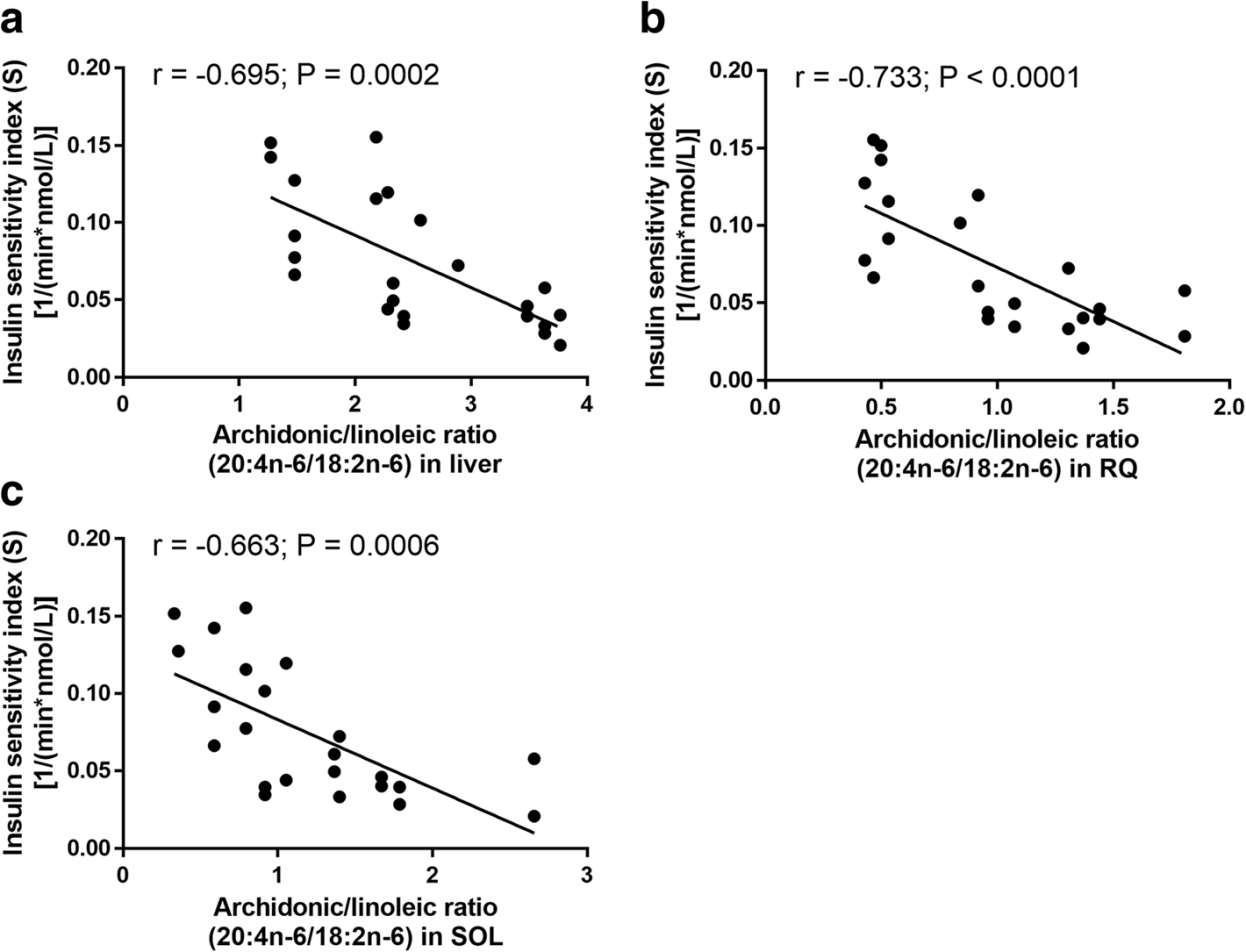
Correlations between arachidonic/linoleic acids (20:4n-6/18:2n-6) ratio of tissue phospholipid and insulin sensitivity in pregnant rats. Correlations between the ratio of the abundance of arachidonic and linoleic acids in liver (**a**), red quadriceps (**b**) and soleus (**c**) muscles with the insulin sensitivity index. Rats from all diet groups were included in this analyses. *N* = 23. RQ, red quadriceps muscle; SOL, soleus muscle

Not surprisingly given the above the ratio 20:4n-6/18:2n-6 (used as a composite index of overall delta-6 desaturase, elongase and delta-5 desaturase activity) was also related to insulin sensitivity in pregnancy, where the 20:4n-6/18:2n-6 ratios in the liver, red quadriceps and soleus were highly, and inversely, correlated to S (*r* = −0.695 for liver, *P* = 0.0002; *r* = −0.733 for red quadriceps, *P* < 0.0001; *r* = −0.663 for soleus, *P* = 0.0006; Fig. 5). Product-precursor relationships are often used to index fatty acid desaturase and elongase activities but one must cautious in pregnancy as fetal drain may skew the analysis.

### Liver triglycerides

In the non-pregnant rats, there was a very modest trend for the n-3 diet to reduce (*P* = 0.29) and the n-6 diet to increase (*P* = 0.11) liver triglycerides as compared to CHOW (Table 1). There was no effect of pregnancy on liver triglycerides in any of the diet groups.

## Discussion

Maternal glucose homeostasis during pregnancy is complex. It is a mixture of both alterations in insulin secretion and insulin action. The overall effect appears to be aimed at ensuring nutrient supply to nurture the fetus plus maternal defence against excessive demand, reflecting the specifics of the drain particularly in glucose and specific fatty acids (e.g. n-3 PUFAs) for nervous system growth. The present study highlights the important role that specific long-chain essential fatty acids can play in these dynamics.

Pregnant rats on the chow diet showed the expected reductions in glycemia and insulin sensitivity and increase in the insulin secretion index compared to non-pregnant chow-fed controls. Diets higher in fat but also increased in either family of long-chain PUFAs had no significant overall effect on insulin secretion and only the n-6 diet resulted in a marginal decrease in insulin sensitivity in non-pregnant rats compared to chow-fed controls (see Fig. 2). In contrast both n-6 and n-3 enriched diets had major effects when the glucoregulatory system is perturbed by the metabolic stress of pregnancy. N-3-enriched diet acted to improve overall glucose homeostasis in pregnancy. This is shown clearly in basal insulin which is seen to be dramatically lowered by n-3 fatty acids and in the insulin AUC to an IVGTT which is markedly lowered with no deterioration in glucose AUC. The calculated measures of insulin sensitivity and secretion demonstrated a complete abrogation of pregnancy-induced changes, maintaining values essentially equal to non-pregnant rats. The effects of the n-6-enriched diet were intermediary between chow and n-3 with evidence, compared to chow controls, of some insulin resistance in the non-pregnant rats but an improvement, again compared to the chow group, during pregnancy.

The nature of the relationship between insulin hyper-secretion and insulin resistance is not straightforward. The traditional argument has been that hyperinsulinemia is a reaction to, or consequence of, hyperglycemia with the high levels of insulin then driving insulin resistance and eventually on to the development of type 2 diabetes [25]. However, this framework for interpreting the etiology of type 2 diabetes has been reevaluated by a number of groups who propose a primary hyperinsulinemia as the critical “lesion” which may trigger the dysmetabolic sequelae leading to type 2 diabetes. Thus it has been well argued that this primary predisposition to excess insulin secretion *per se* is both bad for β-cell health and predisposes insulin resistance [26–30]. This alternative view may be particularly germane to understanding the complex glucose and insulin dynamics of pregnancy. Our data, and that of others, in both rodents [22] and humans [31] suggest that early in pregnancy relative hypoglycemia is evident even as insulin secreting capacity and β-cell proliferation are manifest. Interestingly Holness et al. [32] showed that increased dietary n-3 (on a saturated fat background) in pregnant rats reduced glucose-stimulated insulin secretion in isolated islets. Taken together with our findings, these results suggest that the β-cell enlargement and attendant insulin secreting capacity earlier in pregnancy is not related, and certainly not reactive, to control of hyperglycemia. It is not clear from either human or experimental animal studies whether this primary hyperinsulinemia of pregnancy is beneficial (e.g. as a growth factor for fetal development or to provide necessary capacity to meet the insulin resistance of later pregnancy) or detrimental in term of, as noted above, β-cell health and insulin action later in pregnancy. This is a critical area for future work.

High fat diet feeding (n-6 and n-3) had the expected effects on membrane lipid composition in the non-pregnant animals, with increased n-3 long-chain PUFAs and increased n-6 long-chain PUFAs in the n-3 and n-6 groups respectively. However, pregnancy provoked a quite remarkably different effect on membrane lipid composition between liver and muscle tissues in the n-3 group. In the liver, the main effects of pregnancy were increased n-3 content and an approximately equivalent decreased n-6 content in the n-3 group, the net effect being reflected in a major increase in the n-3/n-6 ratio from 0.46 ± 0.01 to 0.94 ± 0.02. This pattern is consistent with earlier work by Childs et al. [33]. In contrast, pregnancy was associated with a consistent decrease in skeletal muscle n-3 and an increase in n-6 content, largely independent of dietary treatment. There is significant fetal demand for n-3 fatty acids during pregnancy, particularly for nervous system development. It is therefore interesting that, despite this, the liver avidly sequesters even more n-3 fatty acids, a result consistent with those of Cunnane and Armstrong [34] who have previously shown a marked, steady increase in hepatic phospholipid n-3 fatty acids during pregnancy, peaking at partuition and declining to control levels by 9 days post-partum.

The increased levels of n-3 PUFAs in the liver during pregnancy in the n-3 diet group is in line with the function of the liver to distribute n-3 PUFAs in very-low-density-lipoprotein particles to the growing fetus. During pregnancy the fetus requires long-chain n-3 PUFAs, particularly DHA, via the placenta especially for nervous system development. Certainly in humans, during pregnancy, and especially during the last trimester, secretion of very-low-density-lipoprotein (triglycerides) from the liver is markedly increased [35]. There is evidence, at least in rats, of strong correlations between liver phospholipid/triglyceride fatty acid composition and plasma fatty acid profile [36]. The lack of increase in liver n-3 PUFAs during pregnancy in the CHOW and n-6 diet groups may then suggest a relative dietary paucity of n-3 fatty acids works against the ability of the liver to deliver optimal n-3 fatty acids to the fetus.

Muscles are depleted of n-3 fatty acids, presumably to support fetal demand. The reduction in n-3 levels in phospholipid averaged approximately 16.5 % across both muscles in the 3 diet groups. Based on our early work [17] and that of others [37], this is likely to result in a decrease in muscle insulin sensitivity and contribute substantially to pregnancy-induced insulin resistance. The positive, and highly significant relationships between n-3 in phospholipid and insulin sensitivity in all sampled tissues is consistent with this, and highly supportive of the role of n-3 in insulin action. The inverse relationships between insulin sensitivity and arachidonic acid (20:4n-6) were not seen in non-pregnant rats, but powerful with pregnancy (liver and red quadriceps *r* = −0.785 and −0.754 respectively; both *P* < 0.0001). Supply of DHA and arachidonic acid to the fetus is considered important to development (see [6]) and is consistent with the major pregnancy-induced increase in fatty acid desaturase and elongase activity as seen in the present results. These results are consistent with the work of Childs et al. [33] who demonstrated an increase in delta-6 desaturase (FADS2) gene expression during pregnancy which they related to pregnancy-related hormonal changes (particularly progesterone and estradiol). Overall this aspect of our results highlights the fine balance between provision of arachidonic acid to the fetus, its negative effect on insulin action (which may be a part of diversion of nutrients to the fetus) and the relatively pro-inflammatory effects of arachidonic 2-series metabolites. Taken together these considerations strongly emphasize the potentially critical balance of dietary n-3 and n-6 PUFAs intake during pregnancy.

We have previously reported an apparent protective effect of PUFA consumption against the development of glucose intolerance during pregnancy in a sample of Chinese women [13]. Due to limitations in dietary methodologies we were unable in that study to determine whether this effect was related to n-3 or n-6 consumption, since both were present in the major dietary source of PUFAs in this population (soy oil). To the extent that the present study in rats is comparable to the human pregnant state, our results strongly favor the involvement of n-3 fatty acids in this protective effect.

Finally, both high fat diets resulted in increased body weight compared to the chow-fed controls independent of pregnancy status. Thus under conditions of matched caloric intake the animals were able to increase body weight in response to increased dietary fat and to pregnancy alone. Pregnancy in humans has been associated with reductions in free-living physical activity sufficient to nearly account for the increased energy costs of pregnancy [38] as well as increased energy efficiency during treadmill exercise [39]. Our results are consistent with such increases in energetic efficiency.

The experimental diet model used here provides a platform for further investigation of a number of critical questions. First, is hyperinsulinemia early in pregnancy positive or negative for the mother or the fetus? Using the n-3 diet compared to the other two diets the effect of reducing insulinemia, and not impairing glucoregulation, on offspring pancreatic β-cell status at birth and lifespan glucoregulation can be studied. Second, by shifting diets during pregnancy insulinemia can be manipulated over periods corresponding to first, second and third trimesters of pregnancy to determine the effect on maternal glucoregulatory “health” during the latter phases of pregnancy including development of insulin resistance. These results can then inform human trials. Of equal importance in these studies will be the subsequent effects on offspring epigenetic markers, body composition, pancreatic β-cell status and insulin secreting capacity.

## Conclusions

The present results demonstrate that the amount and type of dietary fat has potent effects on insulin secretion and sensitivity during pregnancy. N-3 fatty acid incorporation into structural lipid in liver and muscle positively relates to insulin sensitivity. The benefits of high n-6 PUFA diets are less clear. The greater the unsaturation/elongation of linoleic to arachidonic acid, the greater the insulin resistance in pregnant rats, emphasizing the need to focus on both dietary fat amount and n-3/n-6 PUFA balance in pregnancy. The experimental model described provides a platform to investigate the critical issue of the role of hyperinsulinemia in glucoregulation of both mothers during pregnancy and subsequently in offspring.

## Additional file

Additional file 1: Table S1. Fatty acid composition in skeletal muscle and liver. This dataset included individual fatty acid profile in red quadriceps, soleus and liver of each animal. (XLSX 51 kb)

## Abbreviations

AUC: Area under the curve
DHA: Docosahexanoic acid
GDM: Gestational diabetes
IVGTT: Intravenous glucose tolerance test
PUFAs: Polyunsaturated fatty acids
S: Insulin sensitivity
β: Insulin secretion
